# Supplementary material for: Anti-Inflammatory Effect of Caffeine on Muscle under Lipopolysaccharide-Induced Inflammation
Source: Antioxidants (Basel). 2023 Feb 23;12(3):554. doi: 10.3390/antiox12030554 (PMC10045054; doi:10.3390/antiox12030554)
Supplement: Supplementary file 1 [file antioxidants-12-00554-s001.zip › antioxidants-2200045-supplementary.pdf]

antioxidants

Supplementary Materials

Tuany Eichwald [1], Alexandre Francisco Solano [1], Jennyffer Souza [1], Taís Browne de Miranda [2], Liebert Bernardes Carvalho [2], Paula Lemes dos Santos Sanna [2], Rodrigo A. Foganholi da Silva [2,3,†] and Alexandra Latini [1,*,†]

1 Laboratory of Bioenergetics and Oxidative Stress—LABOX, Department of Biochemistry, Federal University of Santa Catarina, Florianópolis 88037-100, Brazil
2 Epigenetic Study Center and Gene Regulation—CEEpiRG, Program in Environmental and Experimental Pathology, Paulista University—UNIP, São Paulo 05508-070, Brazil
3 School of Dentistry, University of Taubaté, Taubaté 12020-3400, Brazil
* Correspondence: alatinilabox@gmail.com; Tel.: +55-48-3721-4743; fax: +55-48-3721-9672
† These authors contributed equally to this work.

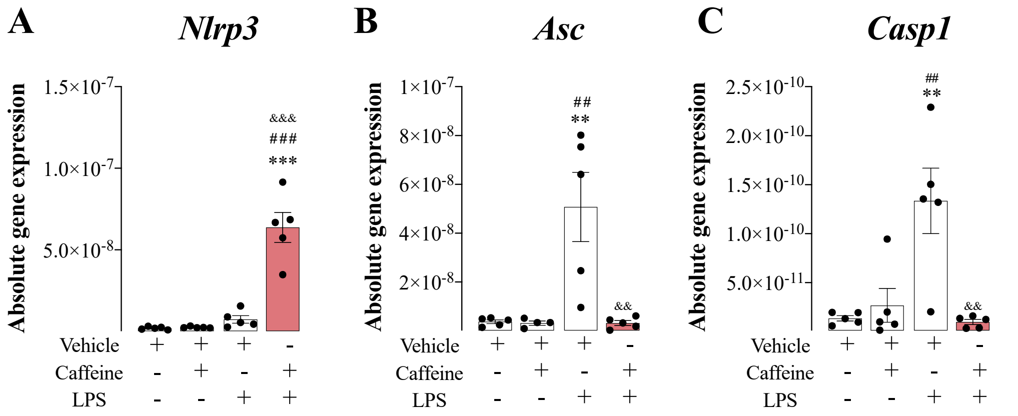

Figure S1. Caffeine administration prevented lipopolysaccharide (LPS)-induced Nrlp3 inflammasome components upregulation in the *vastus lateralis* muscle of mice. Adult Swiss male mice (3–5 months of age; body mass 45-50 g) received a single intraperitoneal (i.p) injection of caffeine and/or LPS (See M&M for details). NLRP3 inflammasome consists of a sensor (Nlrp3), an adaptor (Asc) and an effector (Caspase-1) (A). The absolute gene expression of the components of the NLRP3 inflammasome *Nlrp3* (B), *Asc1* (C), and *Casp1* (D). Bars represent the mean ± standard error of mean of 5 independent experiments (animals) performed in technical duplicates. $^{**}P < 0.01$; $^{***}P < 0.001$ *vs.* vehicle; $^{\#\#}P < 0.01$; $^{\#\#\#}P < 0.001$ *vs.* to caffeine, and $^{\&\&}P < 0.01$; $^{\&\&\&}P < 0.001$ *vs.* LPS. Two-way ANOVA followed by the Tukey's test.

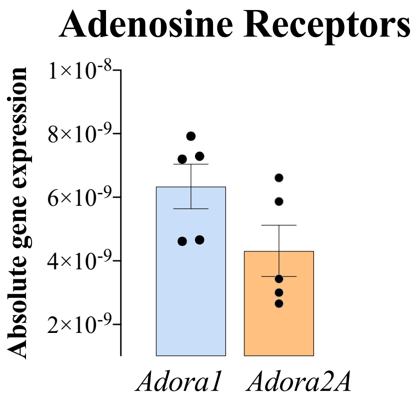

Figure S2. Absolute gene expression of adenosine receptors in the mouse *vastus lateralis* muscle. Adult Swiss male mice (3–5 months of age; body mass 45-50 g). The absolute gene expression of

adesonime receptors *Adora1* and *Adora2A* were evaluated by qPCR after the total RNA extraction (TRIzol®/Chloroform/Isopropanol method) from the muscle. Bars represent the mean ± standard error of mean of 5 independent experiments (animals) performed in technical duplicates. Student's *t*-test.
